# Effect of selenium supplementation on performance, cost economics, and biochemical profile of Nellore ram lambs

**DOI:** 10.14202/vetworld.2015.1150-1155

**Published:** 2015-09-30

**Authors:** K. Sushma, Y. Ramana Reddy, N. Nalini Kumari, P. Baswa Reddy, T. Raghunandan, K. Sridhar

**Affiliations:** 1 Department of Instructional Livestock Farm Complex, College of Veterinary Science, S. V. Veterinary University, Hyderabad - 500 030, Andhra Pradesh, India; 2 International Livestock Research Institute, Patancheru, Hyderabad - 502 324, Andhra Pradesh, India; 3 Department of Animal Nutrition, College of Veterinary Science, S. V. Veterinary University, Hyderabad - 500 030, Telengana, India; 4 National Research Council on Meat, Chengicherla, Hyderabad - 500 092, Andhra Pradesh, India

**Keywords:** biochemical profile, carcass characteristics, Nellore ram lambs, performance, selenium supplementation

## Abstract

**Aim:**

Present experiment was conducted to investigate the effect of selenium (Se) supplementation on performance, carcass characteristics, meat composition, shelflife of meat and biochemical profile in Nellore ram lambs.

**Materials and Methods:**

24 male Nellore ram lambs (15.75±0.47 kg) were randomly divided into four dietary groups with six lambs in each and reared under uniform management conditions for 120 days. Basal diet was not supplemented with Se and consisted of green fodder (Se 0.09 mg/kg dry matter [DM]), dry roughage (Se 0.11 mg/kg DM) and concentrate mixture (Se 0.019 mg/kg DM) and fed individually. Dietary treatments were prepared by adding graded levels Se (0, 0.45, 0.9, and 1.8 ppm) to concentrate mixture (1% body weight [BW]) from sodium selenite. Feed offered and refusal measured daily; and BWs were measured at fortnight interval to find out average daily gain (g), feed conversion ratio (FCR), cost economics and plane of nutrition. Serum biochemical profile (concentration of glucose, total protein, albumin, globulin, cholesterol, and hemoglobin) was assessed on 0, 60^th^, and 120^th^ day. At the end of experiment, the carcass characteristics (dressing percentage, cut-up parts, meat to bone ratio) and meat chemical composition were evaluated. Meat keeping (thiobarbituric acid reactive substances) quality from different groups was evaluated on day 0, 3, and 6 post-slaughter.

**Results:**

Dietary Se supplementation did not show any effect on weight gain, FCR, cost economics, plane of nutrition, and serum biochemical profile in Nellore ram lambs. However, Se supplemented lambs had numerically higher weight gain than the unsupplemented lambs. Similarly, carcass characteristics and keeping quality were comparable among the four treatments. However, numerical increase in post-slaughter keeping quality with increasing Se supplementation was observed.

**Conclusion:**

It can be concluded that supplementation of Se in the form of sodium selenite (inorganic source) at different levels did not influence animal performance in growing Nellore ram lambs had no effect on lamb performance, cost economics, carcass characteristics, and serum biochemical profile.

## Introduction

Sheep and goat provide economic support to the small and marginal farmers and landless laborers in tropical countries which are traditionally managed on grazing lands. However, in open range, rearing system lambs are prone to walk and environmental stress, in addition to nutritional insufficiency, makes the animal more susceptible to various bacterial, viral and parasitic infections. All these conditions cumulatively affect the lamb immunity which will indirectly affect the lamb performance parameters like growth rate, feed intake, feed conversion ratio (FCR) and carcass yield and quality. To maximize the profit, the major goal of sheep farmers is to minimize the losses from diseases and attain good flock viability, which could be obtained by improving the disease resistance capacity of the flock. The ability of sheep to withstand various infectious diseases depends on the integrity of the immune system. The immune system mainly depends on the nutrients availability that mediates cellular functions pertinent to host defense. Nutrition is the major decisive factor in determining the expression of the genetic potential of lambs in terms of growth and immunity [1].

Selenium (Se) is one of the essential trace elements required for maintaining proper health and immunity of lambs. The role of Se in lamb health is based on the functions of selenoproteins, many of which have antioxidant activities [2]. Nutrient requirements of dairy cattle [3] recommended a dietary level of 0.3 ppm of Se for cattle sheep and pigs, whereas, salt institute [4] has recommended 30 ppm Se to be added in the salt mineral mixture for sheep as compared to 20 ppm for cattle, including that Se requirement of sheep might be higher than cattle. Lee *et al*. [5]; Dominguez-Vara *et al*. [6] were observed no effect of additional Se supplementation on performance of steers and lambs, respectively.

Keeping in view the fact that very limited information is available on the effect of Se supplementation on the performance of tropical sheep, present study was conducted to investigate the effect of increasing Se supplementation on growth rate, feed intake, FCR, blood biochemical profile and carcass characteristics and keeping quality in Nellore ram lambs.

## Materials and Methods

### Ethical approval

The experiment was approved by Institutional Ethics Committee of S.V. Veterinary University.

### Site of study

The experiment was conducted at the Department of Animal Nutrition, College of Veterinary Science, Rajedranagar, Hyderabad, Telengana, India. During the period of experiment the ambient temperature and relative humidity values ranged between 28°C and 34°C and 28-32%.

### Experimental diets and lambs rearing

The basal diet was prepared according to ICAR [7] requirements using green fodder (APBN_1_), dry fodder (sorghum), and concentrate mixture which contained Se (DM basis) at 0.09 ppm, 0.11 ppm, and 0.19 ppm, respectively. Concentrate mixture was prepared by incorporating maize (40%), wheat bran (32%), soybean meal (25%), mineral mixture (2%), salt (1%), and vitamin mix (40 g/100 kg). The experimental diets T1, T2, T3, and T4 were supplemented with 0, 0.1, 0.2, and 0.4 g sodium selenite, respectively to the 100 kg concentrate mixture. It had provided 0, 0.45, 0.9, and 1.8 ppm Se to the lambs, respectively. Twenty four Nellore ram lambs aged 3 months with a mean body weight (BW) of 15.45±0.47 kg were randomly divided into four groups of six lambs each in a completely randomized design. All lambs were housed in well-ventilated pens (4 m×3 m) and maintained healthy surroundings and proper cleanliness during the experimental period. They were dewormed at the beginning and middle of the experiment. The four experimental diets (T1, T2, T3, and T4) were randomly allotted to four groups of ram lambs. The respective diets were offered at 9:00 and 15:00 h daily for 120 days. The lambs were offered restricted amount of respective concentrate mixtures (1% of BW), but green fodder and Stover were offered *ad libitum*. Residues, if any, were weighed on the next day morning before offering of feed. The lambs had free access to clean, fresh, and wholesome water throughout the experiment.

### Live weight recording

The experimental lambs were weighed fortnightly using an electronic digital balance after 16 h of feed and water deprivation. The BWs presented in this study was calculated by taking the mean of BW on two consecutive days. Average daily gain (ADG) for individual lambs was calculated by dividing the sum of daily gains with experiment days. The FCR for each lamb was calculated as a ratio of daily feed intake to ADG.

### Chemical analysis

“Terbotherm” and “vapodest” (Garhard, Germany) analyzers based on the micro kjeldahl method (10; procedure no.4.2.02) was used to analyze the nitrogen content in feed and meat samples. Dry matter (DM), ether extract and total ash were estimated by an AOAC method [8]. Neutral detergent fiber (NDF) and acid detergent fiber (ADF) levels were determined by using the method described by Van Soest *et al*. [9]. The NDF and ADF fractions included residual ash. Crude protein (CP) intake was calculated based on CP content of experimental feeds and metabolizable energy intake (ME) of lambs fed experimental diet were calculated based on literature total digestible nutrients values of this department and NRC formula (3).

### Collection of blood and separation of serum and estimation of serum biochemical constituents

On day 0, 60, and 120 blood samples (15 mL) were collected from each lamb in the morning (before feeding and watering) under aseptic conditions through jugular vein puncture. Immediately after blood collection the vials were kept in slant position without disturbing. After 1 h and centrifuged at 825 rcf for 15 min at 4°C to separate out clear serum, which was collected into small plastic vials (2 ml) and stored at −20°C for further analysis of serum biochemical constituents such as serum total protein and albumin [10], cholesterol [11], and glucose [12]. Globulins concentration was calculated by subtracting the values of albumin from total proteins.

### Carcass studies

At the end of growth trial all the lambs were slaughtered by exsanguination using conventional humane procedure after 16 h of feed and water deprivation to study the carcass characteristics. Before slaughter live BWs were recorded. After complete bleeding, the bodies were skinned, and external organs such as head, feed and skin were weighed. Stripping, legging, dressing, and evisceration were performed by adopting the standard procedure described by Gerrand [13]. Hot carcasses and organs weights were recorded. The carcasses were then divided into 5 cuts, legs, loin, rack, neck, and shoulder and fore shanks as suggested by national livestock and meat board of the United States of America [14], and the wholesale cuts weights were recorded separately. Empty BW (EBW) was recorded after deducting gut fill from preslaughter weight. The weights of liver, heart, kidney, testes, spleen, lungs, trachea, full and empty gastro intestinal track, esophagus, skin, blood, and dressed head were expressed as percent of preslaughter weight. The dressing percentage was expressed on preslaughter weight and EBW basis. The cut wise separated bone, meat and fat of each group were weighed expressed as percentages of whole carcass and calculated meat to bone ratio.

### Estimation of carcass keeping quality

Carcass keeping quality was assessed by estimation of thiobarbituric acid (TBA) levels in homogenized meat sample. In this method, 4 g of meat sample was taken in a test tube and 20 mL of 20% trichloro acetic acid was added to the tube. Then, it was homogenized and filtered through the filter paper. The 3 ml of filtrate was added to 2 ml of TBA in a test tube and covered the test tube with aluminum foil. The tube was mixed using vortex mixer and kept in hot water bath for 30 min. The tubes were mixed again using a vortex mixer and readings were measured in UV spectrophotometer at 340 nm for estimating TBA levels.

### Economics

The cost of experimental rations was calculated on the basis of prevailing market price of ingredients and processing cost. The average processing cost for various feeds was calculated based on two shifts of 8 h each 300 working day/year. Direct charges include power consumption, operators, Labor wages, etc. The fixed charges include depreciation on equipment, interest on building, insurance, and the maintenance charges. Total processing cost was calculated by adding fixed charges and direct charges. Thus, the total cost of ration was obtained by summing the cost of processing plus cost of feed consumed throughout the feeding period (120 days). The cost per kg live weight gain was obtained by dividing the total cost of ration consumed with total BW gain.

### Statistical analysis

The data was subjected to one-way ANOVA. The differences between the means were tested using Duncan’s multiple range test [15]. All the statistical procedures were carried out as per the procedures of Snedecor and Cochran [16].

## Results

### Growth study, cost economics and serum biochemical profile

The percent CP, NDF, ADF values of green fodder, dry fodder, and concentrate mixture were ranged from 9.41, 6.52, 15.36; 71.77, 73.51, 30.55; 44.20, 48.20, 20.30% (Table-1). The ADG, DM intake (DMI) (g/d or g/kg w^0.75^), and FCR did not differ significantly among the dietary groups (Table-2). The CP and ME intakes were similar among the experimental lambs supplemented with different levels of Se.

**Table-1:** Chemical composition (% DM)* of experimental feeds.

Parameter	Green fodder	Dry fodder	Concentrate mixture
Proximate principles			
DM	26.50	93.53	95.12
Organic matter	90.23	93.50	89.24
CP	9.41	6.52	15.36
Ether extract	4.98	1.45	4.23
Crude fiber	35.34	35.56	10.76
Nitrogen free extract	40.50	49.97	59.26
Total ash	9.77	6.50	10.39
Cell wall constituents			
NDF	71.77	73.51	30.55
ADF	44.20	48.20	20.30
Hemicellulose	27.57	25.31	13.25
Cellulose	34.50	32.38	21.20
Acid detergent lignin	6.39	7.21	4.28
ME (MJ/kg DM)	8.32	7.50	11.27
Trace element content			
Selenium (ppm)	0.09	0.11	0.19

Each value is the average of triplicate analysis

* On DM basis except for DM; ME=Metabolizable energy, DM=Dry matter, ADF=Acid detergent fiber, NDF=Neutral detergent fiber, CP=Crude protein

**Table-2:** Effect of supplementation of different levels of selenium on the performance and cost economics in growing Nellore ram lambs.

Parameter	Diet				SEM

	T1	T2	T3	T4	
Initial weight (kg)	15.70	15.78	15.63	15.92	0.22
Final weight (kg)	25.42	25.82	26.07	26.30	0.4
Weight gain (kg)	9.72	10.03	10.43	10.38	0.21
Average daily gain (g)	80.97	83.61	86.95	86.53	2.92
Feed intake (g/d)					
Green fodder	725.83	723.54	729.38	736.46	32.84
Dry fodder	387.71	391.67	392.92	396.67	17.68
Concentrate mixture	206.25	210.00	210.00	210.00	9.42
DMI (g/d)	744.63	751.25	753.88	759.13	20.86
DMI (g/kg w0.75)	77.97	78.08	78.30	78.07	0.47
CP intake (g/d)	73.71	74.46	74.69	75.10	2.10
ME intake (MJ/d)	6.51	6.57	6.59	6.63	0.18
FCR (kg/kg gain)	9.20	8.99	8.67	8.77	0.40
Feed cost/kg gain (₹/kg)	93.56	92.08	89.18	90.80	4.14

Each value is the average of six observations. ME: Metabolizable energy, p>0.05, FCR=Feed conversion ratio, CP=Crude protein, DMI=Dry matter intake, SEM=Standard error of the mean

The cost of feeds offered was taken as ₹1.5/kg for green fodder, ₹4/kg for dry fodder and for concentrate was ₹15/kg. The feed cost (₹/kg live weight gain) ranged from 89.2 to 93.6 in the experimental ram lambs fed T1, T2, T3, and T4 rations (Table-2). Among all the groups, cost (₹/kg live weight gain) was numerically highest in ram lambs fed ration T1, followed by T2, T4, and T3 rations.

Concentration of serum biochemical constituents (glucose, total protein, albumin, globulin, cholesterol, and hemoglobin) were not affected by dietary Se supplementation (Table-3).

**Table-3:** Effect of feeding concentrate mixture with different levels of supplementation of selenium on various blood biochemical parameters in growing Nellore ram lambs.

Parameter	Diet					SEM

	Day	T1	T2	T3	T4	
Glucose (mg/dl)	0^th^	61.60	62.72	62.58	60.85	0.38
	60^th^	61.77	62.22	63.43	61.72	2.81
	120^th^	62.59	62.98	63.14	63.58	2.84
Total protein (g/dl)	0^th^	6.11	6.29	6.30	6.40	0.28
	60^th^	6.26	6.27	6.37	6.33	0.02
	120^th^	6.35	6.43	6.40	6.47	0.04
Albumin (g/dl)	0^th^	3.06	3.12	3.17	3.12	0.04
	60^th^	3.15	3.18	3.17	3.27	0.02
	120^th^	3.29	3.30	3.30	3.32	0.03
Globulin (g/dl)	0^th^	3.04	3.17	3.13	3.28	0.05
	60^th^	3.11	3.09	3.19	3.06	0.03
	120^th^	3.06	3.13	3.10	3.15	0.04
Cholesterol (mg/dl)	0^th^	60.00	57.00	60.83	58.83	2.68
	60^th^	61.94	59.68	59.38	60.04	2.72
	120^th^	64.02	64.39	64.35	64.50	0.24
Hemoglobin (g/dl)	0^th^	9.10	10.67	10.03	11.28	0.31
	60^th^	10.39	10.37	10.54	10.80	0.07
	120^th^	10.26	10.26	10.33	10.56	0.07

Each value is the average of six observations. SEM=Standard error mean

### Carcass characteristics and meat quality

The preslaughter weights, empty BW, carcass weights, whole sale cuts and visceral organs yield were not differed significantly (p>0.05) among the dietary treatments (Table-4). Further, no significant (p>0.05) variation was seen in bone and meat yield (in per cent) and their ratios in various wholesale cuts with gradual increase in Se supplementation from 0 to 1.8 ppm in the diet. Similarly, the chemical composition (moisture, protein, fat and ash) was not affected by the Se supplementation at different levels (Table-5).

**Table-4:** Carcass characteristics of ram lambs supplemented with different levels of selenium.

Parameter	Experimental diets				SEM

	T1	T2	T3	T4	
Preslaughter weight (kg)	23.02	25.60	26.13	25.21	1.15
Empty body weight (kg)	20.34	21.44	22.67	22.19	0.99
Hot carcass weight (kg)	11.21	12.22	12.78	12.49	0.31
Dressing percentage					
On pre slaughter weight basis	47.80	48.94	48.72	47.43	2.18
On empty body weight basis	54.65	56.13	53.16	54.16	2.47
Whole sale cuts (% carcass weight)					
Fore shank	15.97	15.63	15.18	14.89	0.09
Neck and shoulder	27.92	27.41	26.45	27.70	0.15
Rack	8.47	7.86	7.51	8.25	0.04
Loin	15.70	15.63	14.48	14.57	0.08
Leg	30.78	31.34	29.73	29.94	0.17
Visceral organs (% preslaughter weights)					
Liver	1.35	1.30	1.16	1.38	0.01
Kidney	0.27	0.24	0.29	0.25	0.00
Heart	0.38	0.36	0.34	0.31	0.01
Testes	1.00	1.02	1.07	0.99	0.01
GIT full	21.16	20.94	20.47	22.09	0.24
GIT empty	7.60	6.48	7.58	7.14	0.08
Spleen	0.46	0.40	0.43	0.35	0.02
Lungs	0.91	0.94	0.88	0.83	0.01
Trachea	0.17	0.16	0.15	0.20	0.00
Skin	10.21	10.14	9.99	9.62	0.07
Head	7.62	7.74	7.72	8.38	0.07
Blood	2.82	2.93	3.10	3.01	0.04
Esophagus	0.22	0.27	0.27	0.28	0.00
Composition of carcass (% of carcass)					
Carcass weight (kg)	11.08	11.96	11.93	11.91	0.53
Meat	6.86	7.53	7.43	7.46	0.33
Bone	3.20	3.49	3.43	3.45	0.15
Fat	1.02	0.94	1.07	1.00	0.05
M-B ratio	2.14	2.16	2.17	2.16	0.10

Each value is the average of six observations. p>0.05, GIT=Gastro intestinal track, SEM=Standard error mean

**Table-5:** Chemical composition of *Longismus dorsi* muscle on fresh basis (%) in Nellore ram lambs supplemented with different levels of selenium in the concentrate mixture.

Parameter	Experimental diets				SEM

	T1	T2	T3	T4	
Moisture	74.96	74.81	74.68	74.41	0.11
Protein	21.64	21.65	21.71	21.73	0.04
Fat	1.45	1.46	1.48	1.49	0.01
Ash	1.94	2.09	2.13	2.38	0.07

Each value is the average of six observations. p>0.05, SEM=Standard error mean

The keeping quality of meat in different groups was assessed by measuring the thiobarbituric acid reactive substances (TBARS) value in meat on day post slaughter 0, 3 and 6. The TBARS values in meat increased (Table-6) until 6^th^ day which is an indication of lipid peroxidation. Additional Se supplementation could not show any beneficiary effect in regulating the lipid peroxidation and improving the keeping quality of meat.

**Table-6:** Effect of supplementation of different levels of selenium on keeping quality of meat measured in terms of TBARS (mg melanaldehyde/kg meat) in growing Nellore ram lambs.

Day	Experimental diets				SEM

	T1	T2	T3	T4	
0^th^ day	0.20	0.18	0.19	0.19	0.01
3^rd^ day	0.32	0.32	0.32	0.31	0.01
6^th^ day	0.61	0.58	0.55	0.55	0.02

Each value is the average of six observations. p>0.05, SEM=Standard error mean

## Discussion

### Performance and cost economics

The gradual increase in Se supplementation from 0 to 1.8 ppm as sodium selenite had no significant (p>0.05) effect on growth performance (total weight gain and ADG), feed intake and FCR (kg DMI/kg gain) of lambs (Table-2). Our results were in consistence with the findings of Lee *et al*. [5] who observed no difference in performance of Han woo steers with increasing levels of Se from 0 to 0.9 mg Se/kg. Similar to present study results, Dominguez-Vara *et al*. [6] noticed no effect of Se addition as Se enriched yeast (0.3 ppm) to basal diet on performance (final BWs and weight gains) of Rambouillet lambs. This might be due to availability of Se through basal diet, such as green fodder (0.09 ppm), dry roughage (0.11 ppm) or concentrate mixture (0.19 ppm) which may meet the nutrient requirement (0.3 ppm) of sheep (NRC, 2001) [3]. So, additional Se supplementation at 0.45, 0.9, 1.8 ppm as sodium selenite could not show significant effect on lambs performance. Concurrence to our results, several authors [6,17-19] observed no effect of additional Se supplementation on growth performance of lambs. In the present study Se supplemented groups had numerically higher weight gains than the unsupplemented groups, but the level of supplementation had no effect on weight gains. But Kumar *et al*. [20] observed significantly (p<0.05) higher growth rate in lambs with Se (0.15 or 0.3 ppm) supplementation than control group (0 ppm); but no significant (p>0.05) differences observed due to level of Se supplementation. Plane of nutrition (CP and ME intakes) was similar among the experimental lambs (Table-2) and the CP and ME intakes met the requirements according to the ICAR [9] recommendations for lambs growing at 100 g/day.

In the present study, no significant difference was observed in feed cost per kg weight gain. Kumar *et al*. [20] reported, feed required per kg BW gain and cost of feed per kg weight gain were less by about 11% and 17% in groups supplemented with Se at 0.15 and 0.30 ppm levels, respectively, as compared to control group, but the differences were not significant (p>0.05).

### Serum biochemical parameters

The biochemical parameters (glucose, total protein, albumin, globulin, cholesterol, and hemoglobin concentrations) were not influenced (Table-3) either by level of dietary Se supplementation (0.45, 0.9 or 1.8 ppm) or by day of blood collection (0, 60 and 120 days). Kumar *et al*. [21] observed that supplementation of Se at 0.15 ppm level either from organic or inorganic source had no effect on the values of serum biochemical parameters like total protein, albumin, globulin, albumin or globulin ratio, and total cholesterol as they remained similar (p>0.05) in all three groups throughout the experimental period. Moreover, all the values were within the normal range. In consistent with our results, Shinde *et al*. [22] indicated that the supplementation of Se (0.3 ppm) had no effect on the biochemical parameters of buffalo caves.

### Carcass characteristics and meat quality

The preslaughter weight, empty BW, hot carcass weight, dressing percentage, visceral organs yield, wholesale cuts proportion (fore shank, neck and shoulder, rack, loin, and leg), proportion of meat, bone and fat in carcass and chemical composition of *Longissimus dorsi* muscle (meat) were not affected (p>0.05) (Tables-4 and -5) by dietary Se supplementation in Nellore ram lambs. Similarly, meat keeping quality (melanaldehyde, mg/kg meat) was also not influenced by dietary Se supplementation (Table-6). Similar to our findings, Vignola *et al*. [23] observed no significant (p>0.05) effect of Se supplementation (sodium selenite, 0.3 or 0.45 ppm) to the basal diet (0.13 ppm) on meat quality (oxidative stability of meat) and carcass yield. Lee *et al*. [5] observed no significant changes in carcass characteristics (yield and composition) of Han woo steers with dietary Se (sodium selenite, 0.9 mg/kg DM) supplementation. Similarly, Juniper et al. [24] observed no significant (p>0.05) difference in meat keeping (TBARS), though increasing the Se supplementation from 0.19 to 0.5 ppm in diets of sheep.

## Conclusion

The results of the present study indicated that the growth rate, feed efficiency, cost economics, blood biochemical profile, carcass characteristics, meat composition, and meat shelf life were not influenced by the supplementation of Se in the form of sodium selenite (inorganic source) at different levels in Nellore ram lambs.

## Authors’ Contributions

KS, YRR, NNR, BR, and TR implemented the study design. KS and KSR recorded the data and analyzed. KS, YRR, NNR, and KSR drafted the manuscript. YRR, NNR, BR, TR, and KS revised the manuscript. All authors read and approved the final manuscript.
